# Urine proteomic signatures of kidney function decline after hospitalization

**DOI:** 10.1172/jci.insight.195577

**Published:** 2025-08-12

**Authors:** Yumeng Wen, Steven Menez, Heather Thiessen Philbrook, Dennis Moledina, Steven G. Coca, Jiashu Xue, James Kaufman, Vernon Chinchillil, Paul L. Kimmel, T. Alp Ikizler, Chi-Yuan Hsu, Tanika Kelly, Ana Ricardo, Jonathan Himmelfarb, Chirag R. Parikh, ASSESS-AKI, TRIBE-AKI, and Kidney Precision Medicine Project consortia

**Affiliations:** 1 Division of Nephrology, Johns Hopkins University School of Medicine, Baltimore, Maryland, USA.; 2 Section of Nephrology, Department of Medicine, Yale School of Medicine, New Haven, Connecticut, USA.; 3 Division of Nephrology, Department of Medicine, Icahn School of Medicine at Mount Sinai, New York, New York, USA.; 4 Division of Nephrology, New York University Grossman School of Medicine and VA New York Harbor Healthcare System, New York, New York, USA.; 5 Department of Statistics, Pennsylvania State University College of Medicine, Hershey, Pennsylvania, USA.; 6 Department of Medicine, George Washington University, Washington, DC, USA.; 7 Division of Nephrology, Vanderbilt University, Nashville, Tennessee, USA.; 8 Kaiser Permanente Division of Research, Oakland, California, USA.; 9 Division of Nephrology, UCSF, San Francisco, California, USA.; 10 Division of Nephrology, Department of Medicine, University of Illinois Chicago, Chicago, Illinois, USA.; 11 Center for Kidney Disease Innovation, Icahn School of Medicine at Mount Sinai, New York, New York, USA.; 12 The ASSESS-AKI, TRIBE-AKI, and Kidney Precision Medicine Project consortia are detailed in Supplemental Acknowledgments.

**Keywords:** Clinical Research, Nephrology, Biomarkers, Chronic kidney disease, Proteomics

## Abstract

BACKGROUND. Urine proteomics may provide mechanistic insights on why patients experience a higher risk of kidney function decline after hospitalization. METHODS. In 174 patients with and without acute kidney injury (AKI) from the Assessment, Serial Evaluation, and Subsequent Sequelae in AKI (ASSESS-AKI) cohort, we used Olink to profile 2783 urinary proteins collected at 3 months after hospitalization and determined their association with estimated glomerular filtration rate (eGFR) decline during median [IQR] of 5.1 [4.0 to 6.0] years follow-up. In 4 independent cohorts, including the Kidney Precision Medicine Project (KPMP), we determined whether proteins were differentially expressed with AKI. We used weighted correlation network analysis to determine proteins’ cellular enrichment in the kidney transcriptome (single-cell and spatial transcriptomics) in patients with AKI receiving research kidney biopsy.RESULTS. We identified 387 and 10 proteins associated with faster and slower eGFR decline, respectively, most of which were differentially expressed in patients at the time of AKI. Among these proteins, 283 (71%) were expressed by kidney cells in participants with AKI from KPMP. The expression formed 3 clusters enriched in the proximal tubule, degenerative tubule and myeloid cells, and stromal cells, and correlated with histopathological features of AKI, such as tubular injury, interstitial inflammation, and fibrosis, respectively.CONCLUSION. Urinary proteins reflecting degenerative tubular injury, inflammation, and fibrosis are associated with eGFR decline in recently hospitalized patients.FUNDING. National Institute of Diabetes and Digestive Kidney Diseases grants U01DK133081, U01DK133091, U01DK133092, U01DK133093, U01DK133095, U01DK133097, U01DK114866, U01DK114908, U01DK133090, U01DK133113, U01DK133766, U01DK133768, U01DK114907, U01DK114920, U01DK114923, U01DK114933, U24DK114886, UH3DK114926, UH3DK114861, UH3DK114915, UH3DK114937, K23DK128358, R01DK128087, and R01DK140717.

## Abstract 2

We identified proteins in the pathways of degenerative tubular cell injury, inflammation, and fibrosis that are associated with long-term eGFR decline after hospitalization for acute illness.

## Introduction

Kidney injury is a common complication during hospitalization. Clinical acute kidney injury (AKI), defined by a sudden increase in serum creatinine (SCr), complicates the illness course in 20% of hospitalized patients (1, 2). Subclinical kidney injury, defined by the elevation of kidney disease biomarkers without an increase in SCr, can also be seen in 10%–17% of hospitalized patients. Both clinical and subclinical AKI are associated with faster long-term kidney function decline (3–5). Despite the increasing prevalence of AKI and the increasing burden of its complications, there is no approved therapy for facilitating recovery from kidney injury. Therefore, deciphering the mechanisms underpinning long-term kidney function decline in patients with acute illness is crucial to address the current gap in care for these patients.

High-throughput proteomic investigations have been shown to identify key pathways underlying adverse clinical outcomes in patients with AKI and patients with chronic kidney disease (CKD) (6–8). These studies have further identified protein markers that can be used to noninvasively measure altered pathways in large cohorts of patients. The majority of the published literature in this area has focused on the plasma proteome, which could be nonspecific to the kidney tissue and subject to reverse causation when plasma protein levels increase due to the decrease in glomerular filtration rate (GFR) (9). Urine proteomic profiling, on the other hand, offers a unique opportunity to identify urinary proteins that are excreted specifically by the kidney tissue, and thus may provide additional insights in pathways associated with adverse clinical outcomes in patients with kidney diseases.

In this study, we hypothesized that urinary proteins associated with long-term kidney function decline after hospitalization would be enriched and expressed by altered kidney cell types, thus providing a link between clinical and subclinical kidney injury and CKD. We performed urine proteomic profiling in recently hospitalized patients to identify urinary proteins associated with long-term kidney function decline. In addition, we integrated proteins after acute illness by performing urine proteomic profiling in recently hospitalized patients and by integrating the proteomic signatures with the kidney tissue transcriptome in patients with AKI.

## Results

### Urinary proteins’ association with longitudinal kidney function decline.

**Figure 1:**
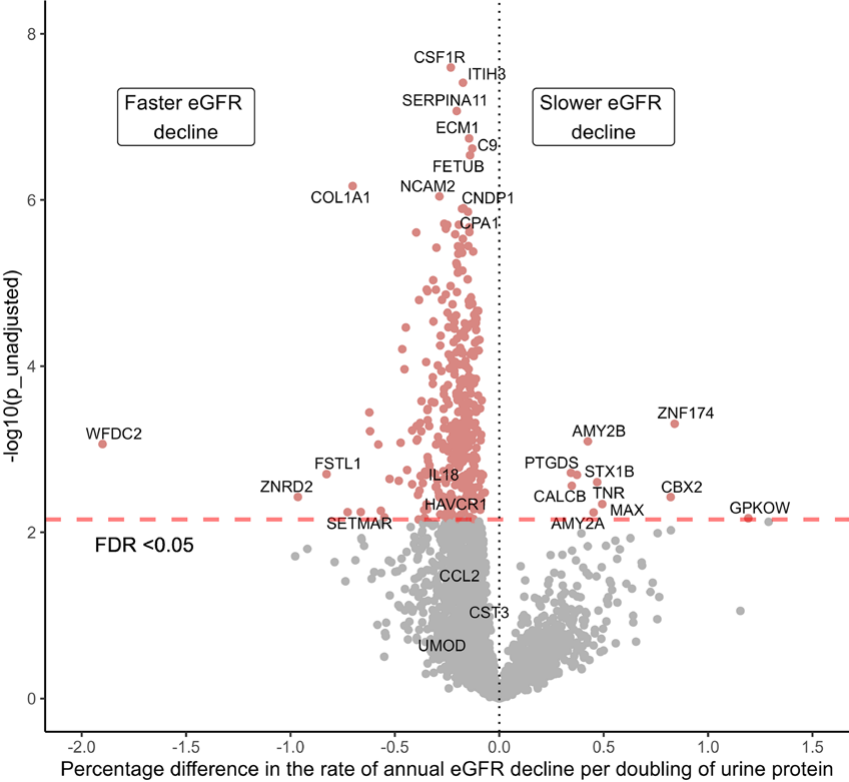
Urinary proteins associated with longitudinal eGFR decline in hospitalized participants with and without AKI from the ASSESS-AKI cohort. The top 10 proteins in the order of significance and the top 5 proteins in the order of effect size for both directions are labeled with gene names. Gray dots represent no significance according to FDR < 0.05.

We used the Olink platform and profiled 2783 urinary proteins using samples collected at 3 months after hospitalization from 174 participants from the Assessment, Serial Evaluation, and Subsequent Sequelae in AKI (ASSESS-AKI) cohort, including 84 and 90 participants with and without AKI, respectively (Table 1) (5). The median [IQR] age was 67.0 [58.7 to 74.6] years and 72 (41.4%) were female. At 3 months after hospitalization, the median [IQR] estimated GFR (eGFR) (using the CKD-EPI 2021 equation; ref. 10) was 65.1 [41 to 84.5] mL/min/1.73 m^2^ and the median [IQR] albuminuria was 16.5 [5 to 158.3] mg. The median [IQR] follow-up time of study participants were 5.1 [4.0 to 6.0] years with a median [IQR] number of 7 [6 to 8] SCr measurements per participant to estimate longitudinal eGFR decline. The mean [IQR] rate of eGFR decline was –0.71% (–0.88% to –0.54%) per year. A total of 397 proteins were significantly associated with eGFR decline (FDR < 0.05, Figure 1). Among the 387 proteins with faster eGFR decline, the top 5 proteins in the order of effect size were WFDC2, ZNRD2, FSTL1, SETMAR, and COL1A1. Known kidney disease biomarkers associated with the AKI-to-CKD transition, such as HAVCR1 and IL-18, were associated with faster eGFR decline but showed smaller effect size (Supplemental Data File 1; supplemental material available online with this article; https://doi.org/10.1172/jci.insight.195577DS1) (11). In addition to FSTL1 and COL1A1, other proteins forming the extracellular matrix material and cell adhesion, such as COL3A1, COL18A1, ECM1, and VCAM1, were also significantly associated with faster eGFR decline (Supplemental Data File 1). A total of 10 proteins were associated with slower eGFR decline; the top 5 proteins in the order of effect size were GPKOW, ZNF174, CBX2, MAX, and TNR. In addition, we identified PTGDS, a marker of podocyte health, that was associated with slower eGFR decline (12).

To determine whether proteins’ association with longitudinal eGFR decline differs in patients with and without clinical AKI, we first analyzed the subgroup of patients with AKI using the primary model, and found that the β coefficients for proteins that are significantly associated with eGFR decline in the entire cohort were similar to results in the AKI subgroup (Supplemental Figure 1). More formally, we tested the interaction between baseline AKI and 397 proteins that were significantly associated with eGFR decline. The interaction term was nonsignificant for the vast majority of proteins (396, or 99.7%), indicating that the effect of these proteins on eGFR decline was similar regardless of baseline AKI. Proteins that were significant in the primary model remained concordantly significant (FDR < 0.05) in the model including the interaction term, further supporting the reliability of their observed associations.

### Urinary proteins associated with kidney function decline are enriched in patients with acute tubular injury.

**Figure 2:**
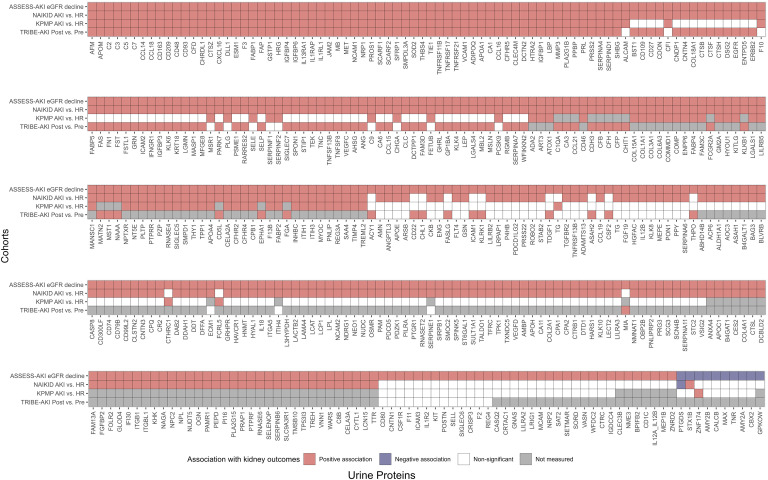
Urinary protein associations with kidney outcomes across cohorts. Positive association (salmon color) indicates that proteins are significantly associated with faster eGFR decline in ASSESS-AKI participants, higher in participants with AKI versus healthy reference in the NAIKID and KPMP cohorts, or higher in post- versus preoperative samples in participants from the TRIBE-AKI cohort. Sorting is based on the number of consistent findings across the 4 cohorts.

Next, we sought to determine whether proteins associated with kidney function decline are enriched in patients with AKI receiving clinical or research kidney biopsies. The first cohort was the Novel Approaches in the Investigation of Kidney Disease (NAIKID) cohort, a prospective cohort enrolling patients with AKI and CKD receiving clinically indicated kidney biopsy and the second cohort was healthy reference participants recruited from Johns Hopkins Hospital. We included 29 patients with biopsy proven acute tubular injury (ATI) and 75 healthy reference participants and had urinary proteins profiled with Olink in the same batch of ASSESS-AKI cohort described above. The age (median, [IQR]) for patients with ATI and healthy reference participants was 60 [49 to 67] and 26 [23 to 33], respectively (Table 2), and 17% and 38% patients with ATI had stage 2 and 3 AKI, respectively. Among the 387 proteins associated with faster eGFR decline in ASSESS-AKI participants, 339 (87.6%) proteins were significantly higher in patients with biopsy-proven ATI (Bonferroni-corrected *P* value < 0.05 [397 proteins analyzed], Figure 2 and Supplemental Figure 2). For proteins associated with slower eGFR decline in ASSESS-AKI, only one protein, PTGDS, was significantly lower in patients with ATI.

The third cohort was the Kidney Precision Medicine Project (KPMP), a prospective cohort of patients with AKI and healthy reference participants receiving research kidney biopsies in the United States (13). We included 29 patients with AKI and 16 healthy reference participants that have urine proteomic profiling with the Somascan V7 platform. The AKI patients were older (48 [22 to 78] vs. 44 [23 to 71] years) than healthy reference. Of those 29 patients with AKI, 6 and 20 participants had stage 2 and 3 AKI, respectively (Table 3). Among the 387 urinary proteins measured by Olink and associated with faster eGFR decline in ASSESS-AKI participants, 321 proteins were measured by Somascan, and 124 were significantly higher in patients with AKI (Bonferroni-corrected *P* value < 0.05 [330 proteins analyzed], Figure 2 and Supplemental Figure 3). One of the 9 proteins associated with slower eGFR decline in ASSESS-AKI and measured by Olink was significantly different between participants with AKI versus healthy reference controls in KPMP.

Lastly, we determined the associations between urinary protein levels and ischemic-reperfusion injury in participants undergoing cardiac surgery from the Translational Investigation of Biomarker Endpoint of AKI (TRIBE-AKI) cohort (Supplemental Table 1). Among the 397 urinary proteins measured by Olink and associated with eGFR decline in ASSESS-AKI, 204 were measured using the Somascan V3 platform in samples collected preoperatively and postoperatively (within 6 hours after surgery) in TRIBE-AKI participants, and 136 were significantly higher after ischemic injury from cardiac surgery (Bonferroni-corrected *P* value < 0.05 [204 proteins analyzed], Figure 2 and Supplemental Figure 4).

### Urinary proteins associated with kidney function decline are expressed in cells of diverse states of health in biopsy tissues from patients with ATI.

**Figure 3:**
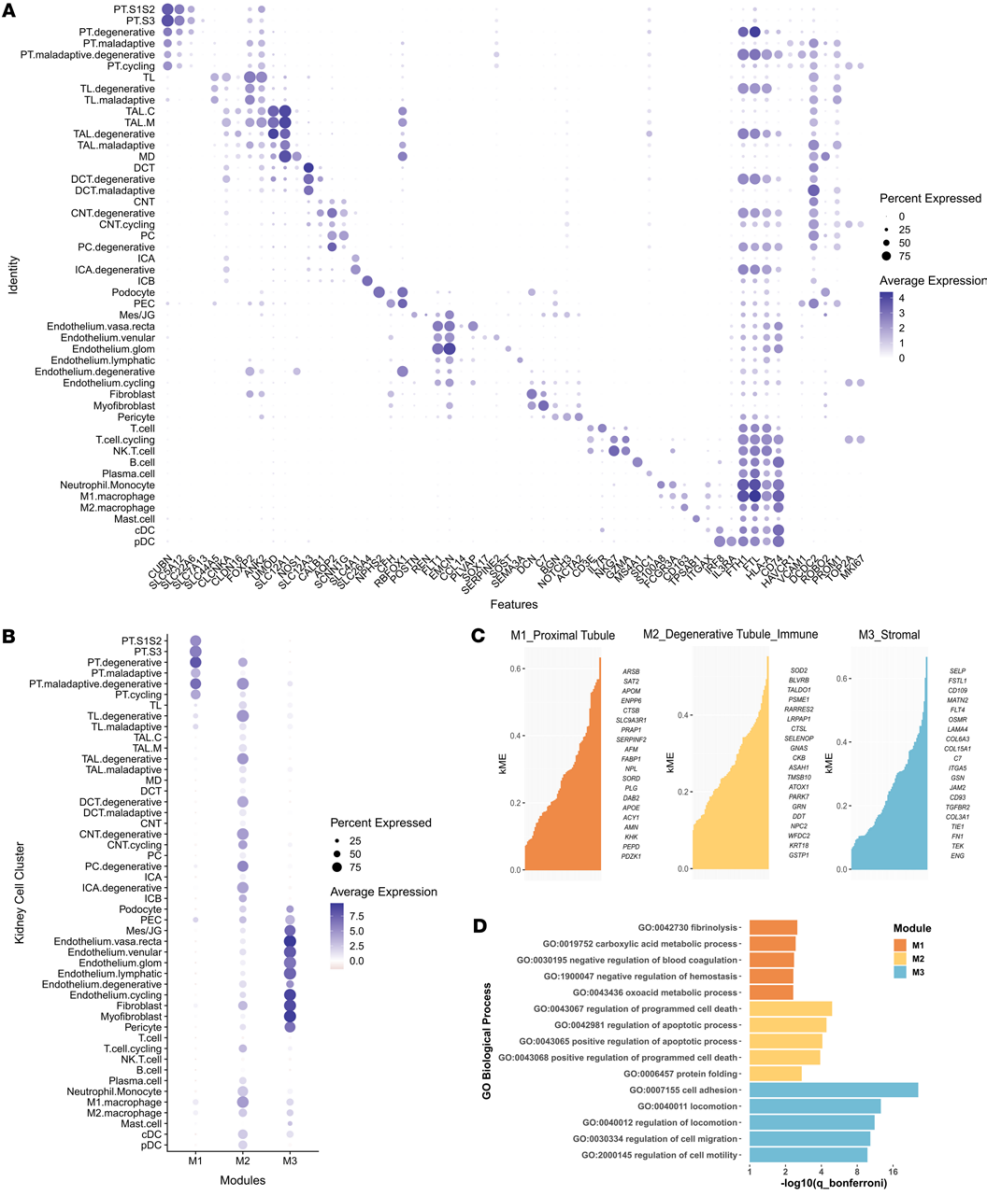
Integration of single-cell transcriptomics and urinary proteins identifies 3 clusters of urinary proteins with genes expressed by diverse kidney cell types in patients with AKI. (**A**) Canonical marker gene expression of major kidney cell types in sc- and snRNA-seq from 20 participants with AKI and 49 healthy reference participants in the KPMP cohort. (**B**) Aggregate expression of gene clusters (modules) in KPMP participants with AKI, using genes with proteins significantly associated with faster or slower eGFR decline from the ASSESS-AKI cohort. (**C**) Top 20 hub genes with the highest eigengene-based connectivity values, representing strong coexpression patterns other genes, within the M1–M3 gene expression module. (**D**) Top 5 Gene Ontology pathways among the M1–M3 gene expression module.

To further gain insights into the mechanisms underlying urinary proteins’ association with kidney function decline, we integrated proteomics findings from the ASSESS-AKI cohort with single-cell and single-nucleus RNA sequencing (sc- and snRNA-seq) data of 279,946 cells/nuclei from 24 participants with AKI and 45 healthy reference controls from the KPMP cohort (Supplemental Data 2). Among participants with AKI, 19 (79%) had stage 2–3 AKI, 12 (50%) had ATI, and 2 (8.3%) had acute interstitial nephritis on their biopsies. The median [IQR] number of cells/nuclei per sample was 2654 [1342 to 4984], and the median [IQR] number of unique molecular identifier per cell/nucleus was 2332 [1320 to 5253]. Using transcriptomic markers identified by our group and others in human and preclinical models of AKI (6, 13–16), we identified renal tubular cells in distinct states of health, as well as major stromal and inflammatory cells (Figure 3A).

Among proteins associated with longitudinal kidney function decline in ASSESS-AKI participants, we first identified 283 proteins with and 114 proteins without genes specifically expressed by any cell type in the kidney in KPMP participants (Supplemental Figure 5). Among the 283 proteins with genes specifically expressed by the kidney, we used weighted correlation network analysis and identified 3 clusters (modules) of proteins whose genes are coexpressed in diverse kidney cell types (Figure 3B). The first module (M1) of genes were coexpressed by the proximal tubular cells and the expression was qualitatively higher in cells in healthy and degenerative state, compared with the maladaptive state (Figure 3B and Supplemental Figure 6). Within this cluster, several genes representing the healthy state of the proximal tubule, such as ENPP6, AFM, and PLG, were among the top 20 hub genes with highest eigengene-based connectivity values, representing strong connection (coexpression) with other genes (Figure 3C) (6). The second module (M2) consisted of genes coexpressed by tubular cells in the degenerative state and myeloid cells, particularly macrophages (Figure 3B and Supplemental Figure 7). Among them, we identified markers of tubular cell injury (WFDC2 and KRT18), and RARRES2, an inflammatory marker of CKD, among the top coexpressed genes (Figure 3C) (17–19). Lastly, the third module (M3) was strongly enriched in stromal cells, including endothelial cells, (myo)fibroblasts, pericytes, and mesangial cells (Figure 3B and Supplemental Figure 8). Consistent with the cellular enrichment, multiple markers of fibrosis (FN1, COL3A1, COL15A1, and COL6A3), cell adhesion (ITGA5) and key components of fibrosis pathways (FSTL1, OSMR, and TGFBR2) formed a close coexpression network (Figure 3C) (20–22). Gene ontologies of these genes further highlight metabolic pathways of the proximal tubule, apoptosis, and cell adhesion and fibrosis for modules M1, M2, and M3, respectively (Figure 3D).

**Figure 4:**
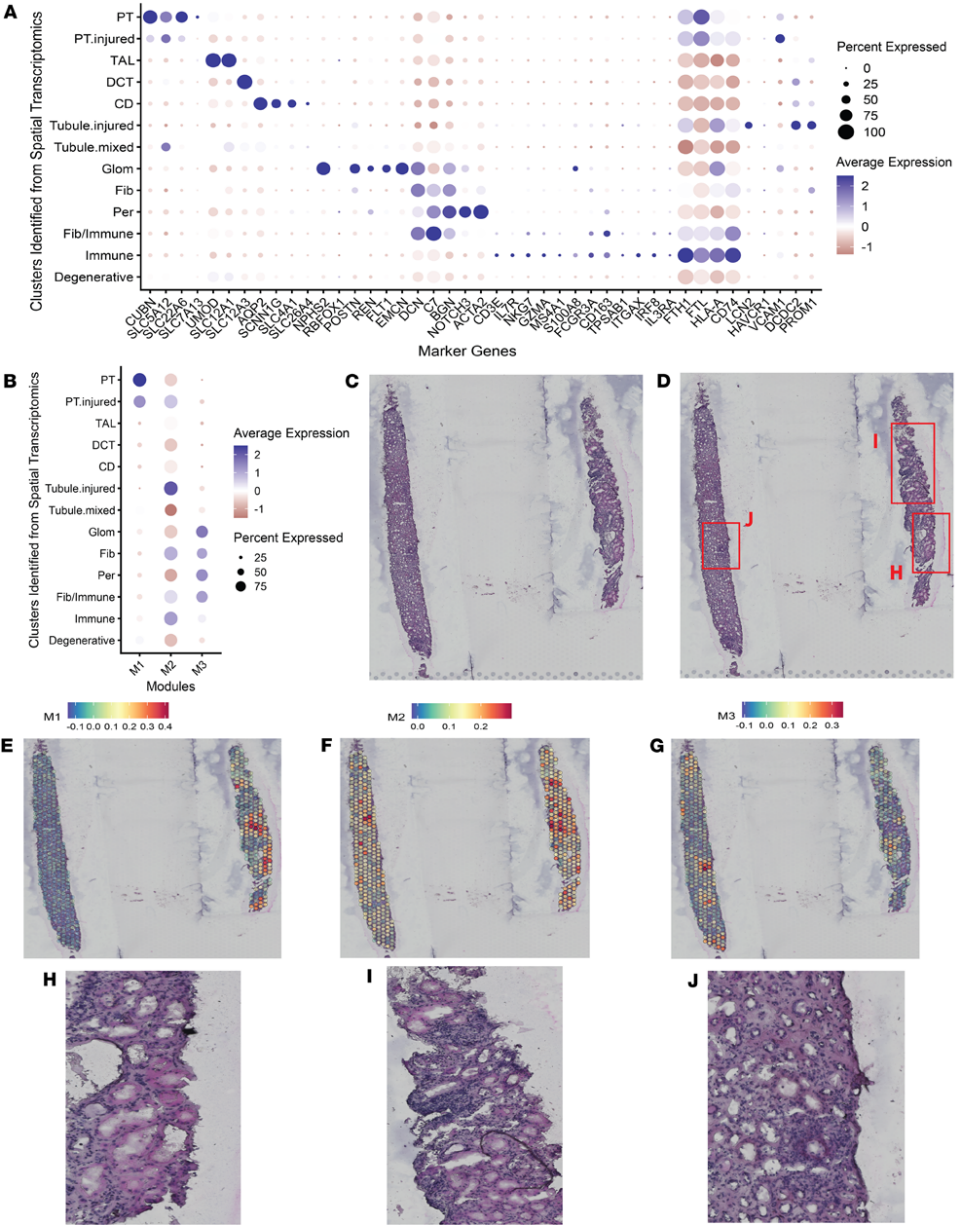
Urinary proteins associated with longitudinal eGFR decline are associated with distinct histopathological features of kidney disease in patients with AKI. (**A**) Canonical marker gene expression of major kidney cell types in Visium spatial transcriptomics from 24 participants with AKI and 7 healthy reference participants in the KPMP cohort. The injured tubular epithelium represents clusters of tubular cells in altered states (maladaptive, degenerative, or the overlap of the two). (**B**) Aggregate expression of gene clusters (modules) in KPMP participants with AKI, using genes with proteins significantly associated with faster or slower eGFR decline from the ASSESS-AKI cohort. (**C**–**J**) Histopathological features of acute tubular injury (**C**, **D**, and **H**–**J**) and heatmaps of the aggregate expression for M1 (**E**), M2 (**F**), and M3 (**G**) gene clusters in one example participant with AKI (participant ID 30-10868) from the KPMP cohort.

Next, we used spatial transcriptomics to validate the expression patterns of these gene modules in the kidney biopsy tissue from 24 participants with AKI and 7 healthy reference controls from KPMP (Figure 4A and Supplemental Data 2). Among these 31 participants, 10 participants with AKI and 3 healthy reference controls were included in the above sc- and snRNA-seq analysis. Although the Visium spatial transcriptomic platform is limited by its lower resolution in terms of cell type clustering, the expression patterns of these gene clusters are highly consistent with findings from single-cell transcriptomics (Figure 4B). In addition, the expression of these clusters was enriched in areas of normal appearing proximal tubule for the M1 cluster, areas of inflammation for the M2 cluster, and fibrosis for the M3 cluster (Figure 4, C–J).

## Discussion

In this study, we performed urine proteomics profiling in recently hospitalized adults with and without clinical AKI, and identified 397 proteins associated with longitudinal eGFR decline. The majority of these proteins were associated with ATI in patients with AKI undergoing kidney biopsies. By integrating proteomic findings with the kidney tissue transcriptomic signatures in patients with AKI, we identified 3 clusters of proteins with genes coexpressed by kidney cells of diverse states of health, highlighting the association between tubular injury, inflammation, and fibrosis and long-term kidney function decline after hospitalization.

AKI is a well-established risk factor for long-term kidney function and the development of CKD (23). Despite the increasing prevalence of AKI, no drugs have demonstrated efficacy in promoting AKI recovery and preventing it from transitioning to CKD. Therefore, there is a high unmet need to discover pathways underlying the AKI-to-CKD transition to facilitate therapeutic development. Renal tubular epithelial cells are the primary site of injury in most cases of AKI during hospitalization (1). The majority of the injured tubular epithelium can repair fully. However, a subsequent proportion of injured cells enter a senescent phase and mediate tubulointerstitial inflammation, fibrosis, and long-term kidney function decline (14). Although kidney function recovery may occur immediately or soon after tubular injury, the recovery course of injured kidney tissue may take weeks to months after the initial insult (24). In addition, subclinical tubular injury, defined by the elevation of biomarkers of tubular injury and inflammation without an increase in SCr, can be seen in 10%–17% of hospitalized patients and is also associated with long-term kidney function decline (3–5). In this study, among urinary proteins measured at 3 months after hospitalization and associated with faster eGFR decline in ASSESS-AKI participants, more than 60% were elevated in patients with biopsy-proven ATI, further suggesting an unresolved signature of tubular injury after hospital discharge.

To delineate mechanisms underlying adverse kidney outcomes, high-throughput quantitative proteomic profiling has been performed in a variety of populations to identify markers of kidney function traits, such as eGFR and albuminuria (7, 25). Integrating proteomic findings with genotypic and kidney tissue transcriptomic information has allowed the identification of key pathways and cell states underlying adverse clinical outcomes in the general population and in patients with acute and chronic kidney diseases (6–9). The majority of the published literature in this area has focused on investigating the plasma proteome, which could be nonspecific to the kidney. The associations between protein concentrations and kidney disease outcome may also be subject to reverse causation, when the plasma proteins increase due to a decrease in glomerular filtration. Urinary proteins, however, may be directly excreted by the kidney tissue, and thus may provide additional mechanistic insights into adverse clinical outcomes in patients with kidney diseases. Although urinary protein concentrations may be affected by urine dilution, studies from our group and others demonstrated the robustness of using regression models and adjusting for urinary creatinine concentration or urinary osmolarity to account for the variation in urinary concentration (8, 26).

We could conceptualize 3 mechanisms for urinary proteins’ association with kidney function decline. First, filtered proteins that are reabsorbed by the renal tubule under healthy conditions could be excreted when tubular function is impaired after injury. Among the 387 proteins associated with longitudinal kidney function decline, approximately 30% were not specifically expressed by any kidney cell types in biopsy tissue from patients with AKI. Thus, their association with kidney function decline may reflect underlying tubular cell dysfunction after AKI. Second, proteins could be secreted by altered kidney cell types that directly mediate decline in kidney function after injury. We identified multiple markers of tubular injury, inflammation, and fibrosis that are associated with longitudinal kidney function decline. The expression of these proteins was clustered in diverse kidney cell types and states, particularly renal tubular cells in degenerative states, myeloid cells, and fibroblasts, further providing evidence for the etiological association between these cell types with adverse kidney outcomes. Genes that have the highest connectivity and coexpression patterns with other genes in the same cluster may be important regulators governing these altered cell states, and thus may be targeted to prevent the AKI-to-CKD transition. Lastly, we identified a cluster of proteins whose genes are enriched in proximal tubular cells at healthy and degenerative states. Within this cluster, we previously reported ENPP6, PLG, and AFM as markers of proximal tubule in healthy states (6). Their plasma concentration decreased after ischemic injury from cardiac surgery and was inversely associated with AKI. The opposite and positive association between the urinary concentration of these proteins with faster eGFR decline may be due to the release of previously synthesized proteins into the urinary space after proximal tubular cell death. Persistent injury, inflammation, and fibrosis have long been investigated as important pathways underpinning kidney disease progression after hospitalization. These results not only validated previous findings using more granular proteomic data, but also provide potential therapeutic targets to mitigate long-term kidney function decline after clinical and subclinical kidney injury.

We recognize several limitations of this study. Our study is based on cohorts with varying sample size and statistical power. Despite this, the directionality of proteomic associations with outcomes are largely consistent. The primary cohort investigating proteomic association with long-term outcome was different from the cohorts for transcriptomic interrogation and validation for proteomic findings, and the assessments were performed at different time points (3 months after hospitalization in ASSESS-AKI vs. at the time of AKI biopsy in KPMP and NAIKID). Due to the small sample size in our kidney biopsy cohorts, we were not able to perform statistically robust analyses with long-term clinical outcomes. In addition, we cannot rule out any discrepancies in the findings due to the difference in time points of sample collection. However, recent transcriptomic studies of AKI and CKD suggest that there is substantial overlap in altered cell states in response to injury in diseased conditions (24). Altered cell states and relevant biological processes may persist months after initial acute injury to the renal tubular cells, providing rationale to use biopsy findings at the time of AKI to aid interpretation of proteins’ association with long-term kidney outcomes after acute illness. Although we controlled for protein variations introduced by age, sex, and urinary concentration, we cannot rule out potential unaccounted confounding considering the difference in demographics between cases and controls in the KPMP and NAIKID cohorts. The ASSESS-AKI cohort is limited by the lack of adjudication or kidney biopsies to discern the etiologies of AKI. Therefore, we were not able to determine whether the urinary proteome and its association with long-term kidney function decline were different between prerenal azotemia and other intrinsic causes of AKI, such as ATI and acute interstitial nephritis. The mapping of urinary proteins to the kidney tissue transcriptome is limited to proteins measured by the proteomic platform, which does not capture the whole proteome. Therefore, this approach may lead to bias when inferring pathways associated with kidney function decline. The identification of proximal tubule cluster may be contributed by overrepresentation of the proximal tubule epithelial cells in the kidney transcriptome, as compared with other cell types. Tubular dysfunction as a potential mechanism underlying proteins’ association with outcomes was inferred based on the lack of kidney transcriptomic enrichment rather than direct functional investigation. However, due to the lack of transcription in the kidney tissue, these proteins may provide limited utility to determine pathways and cell types mediating adverse kidney outcomes.

In summary, integrative investigation of the urinary proteome and kidney transcriptome demonstrated that persistent degenerative tubular cell injury, inflammation, and fibrosis may mediate the long-term kidney function decline in recently hospitalized patients.

## Methods

### Sex as a biological variable.

Male and female patients were used in this study. We used multivariable linear regression to study age, sex, and urinary creatinine for protein variations, and we compared the outcome (AKI during hospitalization, AKI, and ATI) by sex.

### Study population.

The ASSESS-AKI study is a prospective cohort study comprised of 1538 hospitalized adults with and without AKI (1:1 matched) enrolled between December 2009 and February 2015 from 4 North American clinical centers involving various hospital settings (5). The study design has been previously described in detail (5, 27). Briefly, 769 participants who developed AKI and 769 participants without AKI were enrolled during hospitalization. AKI was defined as an increase in SCr concentration of 0.3 mg/dL or more, or at least 50% from the nearest SCr value obtained from an outpatient, non–emergency department setting within 365 days prior to hospitalization (baseline SCr). Participants had their first outpatient research study visits 3 months after discharge. Follow-up study visits were conducted annually thereafter with telephone contacts conducted at 6-month intervals. This subcohort of 174 participants was selected from the ASSESS-AKI cohort as participants who did (*n* = 87) or did not (*n* = 87) develop CKD incidence, progression, or end-stage renal disease in follow-up and was limited to participants with urine samples available. The subcohort includes 84 and 90 participants with and without clinical AKI during their hospitalization, respectively.

We validated proteomics findings from the ASSESS-AKI cohort using samples and data from the KPMP, NAIKID, Hopkins Healthy Reference, and TRIBE-AKI cohorts. To determine whether proteins associated with kidney function decline are enriched in patients with AKI receiving clinical or research kidney biopsies, we included participants and data from the NAIKID, Hopkins Healthy Reference, and KPMP studies.

The NAIKID cohort is an ongoing prospective cohort study of adults who underwent clinically indicated kidney biopsy for the evaluation of acute or chronic kidney diseases. NAIKID participants with biopsy-confirmed ATI were included in this study. The Hopkins Healthy Reference cohort is comprised of healthy participants recruited at the Johns Hopkins Hospital.

KPMP is a National Institute of Diabetes and Digestive Kidney Diseases–sponsored (NIDDK-sponsored) ongoing prospective observational cohort study of participants with AKI and CKD receiving kidney biopsies (28). Participants with AKI were recruited if they developed AKI during hospitalization and had a baseline eGFR of less than 45 mL/min/1.73 m^2^. Biopsies were obtained from total of 40 hospitalized participants with AKI who consented to research biopsies at 4 recruitment sites across the United States: Johns Hopkins Hospital, Yale New Haven Hospital, University of Pittsburgh Medical Center, and Columbia University Medical Center. Additional biopsies were obtained from 4 hospitalized participants with COVID-2019–associated AKI at Johns Hopkins Hospital. Healthy reference tissues were obtained from nontumor regions of kidney tissue after tumor nephrectomy or intraoperative kidney biopsy in participants undergoing urological procedures for nephrolithiasis removal.

The TRIBE-AKI cohort is a longitudinal prospective cohort study of adults who underwent cardiac surgery in 6 academic centers in North America from July 2007 to December 2010 (29). Patients were recruited before cardiac surgery if they were at high risk of developing postoperative AKI and were prospectively followed from enrollment until death, loss to follow-up, or development of end-stage renal disease.

### Urine sample collection and proteomic profiling.

Urine samples were collected in the morning of research visits at 3 months after hospitalization for ASSESS-AKI participants, at the time of kidney biopsy for KPMP participants and for NAIKID participants with ATI, preoperatively and postoperatively (within 6 hours after cardiac surgery) in TRIBE-AKI participants, and in the morning of the research visit for Hopkins Healthy Reference participants. Proteomic profiling was performed using the Olink Explore 3072 platform on urine samples from the ASSESS-AKI, NAIKID, and Hopkins Healthy Reference cohorts. From the 2944 initial proteins, we excluded proteins according to Olink’s QC protocol (143 proteins), proteins where all samples were below the limit of detection (5 proteins), and duplicate proteins (13), resulting in 2783 proteins included in the analysis. Urine samples from the TRIBE-AKI and KPMP cohorts underwent proteomic profiling using the Somascan V3 and V7 platform, respectively. Urinary protein levels underwent standard quality control by Olink and Somascan.

### Statistics for urinary proteins’ association with longitudinal kidney function decline.

We used multivariable linear regression to regress out age, sex, and urinary creatinine, which accounted for protein variations introduced by effects from age, sex, and variation in urinary concentration (7, 26). The residuals were considered adjusted protein levels and used for downstream analysis. We used linear mixed-effects models with random intercepts and slopes to determine the associations between adjusted protein levels and longitudinal decline in eGFR (calculated using the CKD-EPI 2021 equation for creatinine) (10). In these models, we adjusted for baseline CKD status (eGFR < 60 mL/min/1.73 m^2^) prior to hospitalization, AKI status during hospitalization; eGFR, heart failure, diabetes, and hypertension at 3 months; time, and time-protein interaction. We log_2_ transformed eGFR and converted the coefficients of time-protein interaction to represent change in the eGFR decline rate (percentage per year) per doubling of protein concentration. We adjusted for multiple comparisons using the Benjamini-Hochberg method, and considered an FDR of less than 0.05 statistically significant. To evaluate the effect of AKI during hospitalization, we performed 2 sensitivity analyses. We first reproduced the linear mixed-model results among participants with AKI and compared the protein β coefficients with the primary results from all participants. Separately, we examined the significance of interaction terms of AKI and protein in the linear mixed models for all participants.

For validation with the NAIKID cohort, we compared the adjusted protein level between NAIKID participants with biopsy-proven ATI and participants from the Hopkins Healthy Reference cohort using logistic regression. For validation in KPMP and TRIBE-AKI cohorts, proteins measured by Somascan were indexed by urinary creatinine to account for variations in urinary concentration. We compared the log_2_-transformed protein-creatinine ratio between KPMP participants with AKI and healthy reference using 2-tailed Student’s *t* tests, and compared the log_2_-transformed protein-creatinine ratio between post- and preoperative samples in TRIBE-AKI participants using paired Wilcoxon’s tests. We accounted for multiple comparisons in the validation cohorts using Bonferroni’s correction, and considered adjusted *P* values less than 0.05 statistically significant.

### Human transcriptomic dataset library preparation, processing, and analysis.

We used the Cell Ranger 7.0 pipeline (https://www.10xgenomics.com/support/software/cell-ranger) to align sc- and snRNA-seq FASTQ files to the human hg38 reference genome. We then used CellBender to remove ambient RNA contamination and DoubletDetection to remove doublets (30, 31). We used Seurat v4 for data preprocessing and analyses, including normalization, scaling, clustering, dimension reduction, and examination of differential gene expression (32). For snRNA-seq data, after removing ambient RNA contamination and removing doublets, we excluded low-quality nuclei with less than 200 or more than 7500 genes detected. We removed unique molecular identifiers mapped to mitochondrial RNA from analysis and combined all samples for further processing. For scRNA-seq data, we excluded low-quality cells with less than 500 or more than 5000 genes detected and nuclei with greater than 50% mitochondrial reads per cell (13).

We aggregated 200 highly variable genes from each sample and performed log normalization, scaling, principal component analysis, and corrected for batch effects using Harmony (33). We chose the 20 principal components determined by using the ElbowPlot function in Seurat. We further performed dimension reduction to a uniform manifold approximation and projection (UMAP) plot and performed clustering using a resolution of 0.4 after KNN embedding. We annotated major kidney cell types using canonical marker genes identified by the KPMP kidney atlas study (13). Each major cell type underwent subclustering to further remove doublets, which are defined as the expression of canonical marker genes for more than one major kidney cell type (e.g., nuclei or cells expressing both proximal tubule marker CUBN and thick ascending limb marker UMOD) (15). We repeated these steps iteratively until no subcluster of doublets could be identified (15).

For spatial transcriptomic data, we obtained data from the KPMP public data repository. We used sctransform for data normalization and followed the work flow recommended by the Seurat development team, and annotated kidney cell types using canonical marker genes identified by the KPMP kidney atlas study (13).

### Deciphering mechanisms underlying urinary proteins associated with kidney function decline.

We focused our investigation on proteins that were significantly associated with longitudinal kidney function decline in ASSESS-AKI participants. We first used differential gene expression in sc- and snRNA-seq data to identify genes enriched in different cell types in the kidney using Wilcoxon’s tests. We focused on proteins that have genes specifically expressed by the kidney to delineate the mechanisms, such as relevant pathways and cell phenotypes, underlying proteins’ association with kidney function decline. We used weighted correlation network analysis to identify clusters of proteins with genes expressed by kidney cells in sc- and snRNA-seq data from KPMP participants with AKI (34). Pathway enrichment in the gene expression clusters (modules) was explored using ToppFun (35). Within each gene coexpression module, we further determined the top 20 hub genes by their eigengene-based connectivity values. We visualized the expression of these modules in scRNA-seq, scRNA-seq, and spatial transcriptomic datasets to determine whether these clusters were enriched in cells/nuclei in altered states. Using the spatial transcriptomic datasets, we further determined whether these gene clusters’ expression was localized in regions with histopathological features of AKI (36).

### Study approval.

The TRIBE and ASSESS studies (IRB00169832), the KPMP study (IRB00205328), the Hopkins Healthy Reference study (IRB00199993), and the NAIKID study (IRB00221958) were approved by the Johns Hopkins University IRB. Written informed consent was obtained for the use of these human samples.

### Data availability.

Data for Figure 1 and Table 3 are reported in the Supporting Data Values file. Data for findings from the ASSESS-AKI study can be requested from the NIDDK Central Repository and for the KPMP study are publicly available through the KPMP atlas or after request to the KPMP consortium with a Data Use Agreement. The TRIBE-AKI study, Hopkins Health Reference study, and Hopkins NAIKID study are consented observational research studies that are not publicly available but may be available upon reasonable request to the corresponding author with the completion of study regulatory requirements.

## Author contributions

YW, SM, DM, SGC, JK, VC, PLK, TAI, CYH, TK, AR, JH, and CRP assisted with study design of the 4 cohorts. YW, JX, and HTP accessed and verified the underlying data and performed analysis. SM, JK, VC, PLK, TAI, CYH, TK, AR, and JH read the draft manuscript for detailed editorial feedback. CRP provided funding, study design, and writing and editing the manuscript. YW, DM, SGC, and HTP drafted the manuscript.

## Supplementary Material

Supplemental data

Supporting data values

## Figures and Tables

**Table 1:**
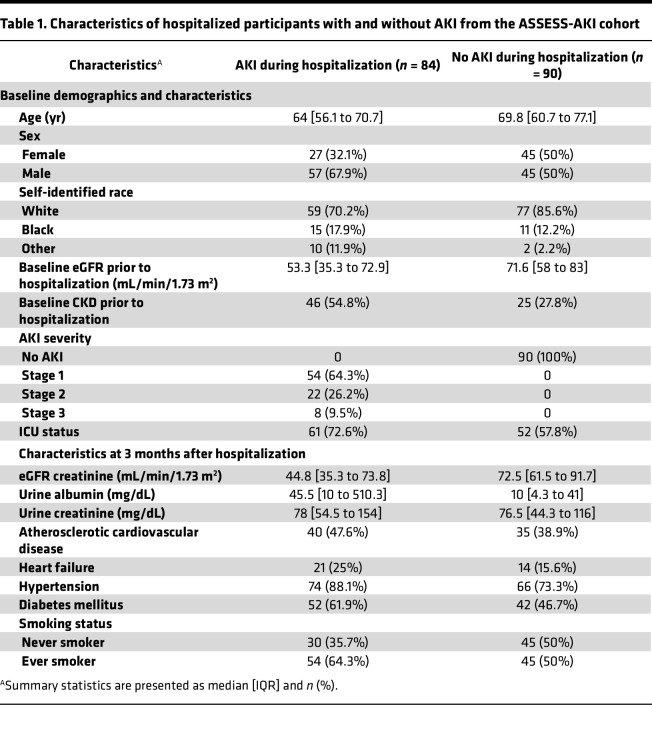
Characteristics of hospitalized participants with and without AKI from the ASSESS-AKI cohort

**Table 2:**
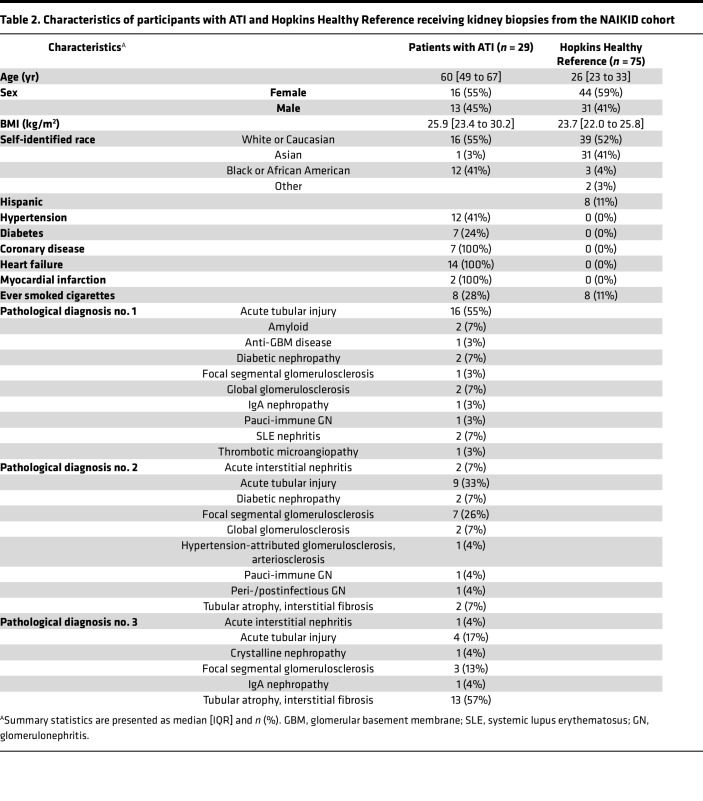
Characteristics of participants with ATI and Hopkins Healthy Reference receiving kidney biopsies from the NAIKID cohort

**Table 3:**
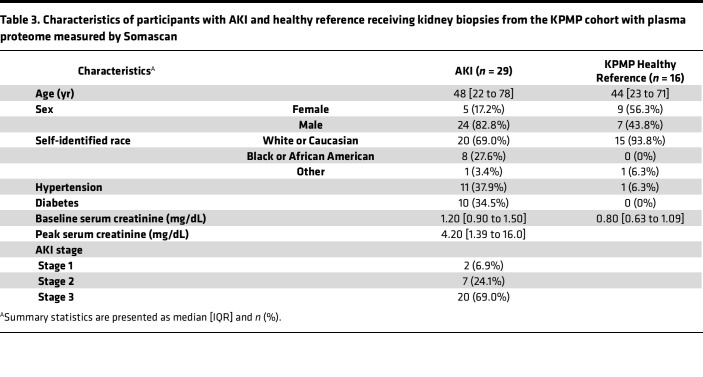
Characteristics of participants with AKI and healthy reference receiving kidney biopsies from the KPMP cohort with plasma proteome measured by Somascan

## Version 1 08/12/2025

In-Press Preview

## Version 2 09/23/2025

Electronic publication

## References

[1] Ronco C (2019). Acute kidney injury. Lancet.

[2] Chawla LS (2014). Acute kidney injury and chronic kidney disease as interconnected syndromes. N Engl J Med.

[3] Molinari L (2022). Utility of biomarkers for sepsis-associated acute kidney injury staging. JAMA Netw Open.

[4] Dépret F (2020). Incidence and outcome of subclinical acute kidney injury using penKid in critically ill patients. Am J Respir Crit Care Med.

[5] Hsu CY (2020). Post-acute kidney injury proteinuria and subsequent kidney disease progression: the assessment, serial evaluation, and subsequent sequelae in acute kidney injury (ASSESS-AKI) study. JAMA Intern Med.

[6] Wen Y (2023). Analysis of the human kidney transcriptome and plasma proteome identifies markers of proximal tubule maladaptation to injury. Sci Transl Med.

[7] Grams ME (2021). Proteins associated with risk of kidney function decline in the general population. J Am Soc Nephrol.

[8] Schmidt IM (2024). Plasma proteomics of acute tubular injury. Nat Commun.

[9] Schlosser P (2023). Transcriptome- and proteome-wide association studies nominate determinants of kidney function and damage. Genome Biol.

[10] Inker LA (2021). New creatinine- and cystatin C-based equations to estimate GFR without race. N Engl J Med.

[11] Wen Y, Parikh CR (2021). Current concepts and advances in biomarkers of acute kidney injury. Crit Rev Clin Lab Sci.

[12] Chen Q (2023). Cell-type-specific molecular characterization of cells from circulation and kidney in IgA nephropathy with nephrotic syndrome. Front Immunol.

[13] Lake BB (2023). An atlas of healthy and injured cell states and niches in the human kidney. Nature.

[14] Kirita Y (2020). Cell profiling of mouse acute kidney injury reveals conserved cellular responses to injury. Proc Natl Acad Sci U S A.

[15] Hinze C (2022). Single-cell transcriptomics reveals common epithelial response patterns in human acute kidney injury. Genome Med.

[16] Xu L (2022). Immune-mediated tubule atrophy promotes acute kidney injury to chronic kidney disease transition. Nat Commun.

[17] Liu L (2020). Integrative informatics analysis of transcriptome and identification of interacted genes in the glomeruli and tubules in CKD. Front Med (Lausanne).

[18] Viñas JL (2020). Sex diversity in proximal tubule and endothelial gene expression in mice with ischemic acute kidney injury. Clin Sci (Lond).

[19] El-Tahir F (2024). Chemerin, TNF - α and the degree of albuminuria in patients with diabetic kidney disease. Cytokine.

[20] Kokkotis G (2024). Oncostatin M induces a pro-inflammatory phenotype in intestinal subepithelial myofibroblasts. Inflamm Bowel Dis.

[21] Li X (2021). Targeting FSTL1 for multiple fibrotic and systemic autoimmune diseases. Mol Ther.

[22] Kökény G (2021). PPARγ and TGFβ-major regulators of metabolism, inflammation, and fibrosis in the lungs and kidneys. Int J Mol Sci.

[23] Coca SG (2012). Chronic kidney disease after acute kidney injury: a systematic review and meta-analysis. Kidney Int.

[24] Zhao ZB (2023). Tubular epithelial cell HMGB1 promotes AKI-CKD transition by sensitizing cycling tubular cells to oxidative stress: a rationale for targeting HMGB1 during AKI recovery. J Am Soc Nephrol.

[25] Kiernan E (2023). Alterations in the circulating proteome associated with albuminuria. J Am Soc Nephrol.

[26] Wen Y (2022). Considerations in controlling for urine concentration for biomarkers of kidney disease progression after acute kidney injury. Kidney Int Rep.

[27] Ikizler TA (2021). A prospective cohort study of acute kidney injury and kidney outcomes, cardiovascular events, and death. Kidney Int.

[28] De Boer IH (2021). Rationale and design of the kidney precision medicine project. Kidney Int.

[29] Parikh CR (2011). Postoperative biomarkers predict acute kidney injury and poor outcomes after adult cardiac surgery. J Am Soc Nephrol.

[30] https://github.com/JonathanShor/DoubletDetection.

[31] Fleming SJ (2023). Unsupervised removal of systematic background noise from droplet-based single-cell experiments using CellBender. Nat Methods.

[32] Hao Y (2021). Integrated analysis of multimodal single-cell data. Cell.

[33] Korsunsky I (2019). Fast, sensitive and accurate integration of single-cell data with Harmony. Nat Methods.

[34] Morabito S (2023). hdWGCNA identifies co-expression networks in high-dimensional transcriptomics data. Cell Rep Methods.

[35] Chen J (2007). Improved human disease candidate gene prioritization using mouse phenotype. BMC Bioinformatics.

[36] Wen Y (2020). A systematic review of clinical characteristics and histologic descriptions of acute tubular injury. Kidney Int Rep.

