# Supplementary material for: Urine proteomic signatures of kidney function decline after hospitalization
Source: JCI Insight. 2025 Aug 12;10(18):e195577. doi: 10.1172/jci.insight.195577 (PMC12487845; doi:10.1172/jci.insight.195577)
Supplement: Supplemental data [file jciinsight-10-195577-s235.pdf]

Urine Proteomic Signatures of Kidney Function Decline after Hospitalization - Supplemental Material

Yumeng Wen[1], Steven Menez[1], Heather Thiessen Philbrook[1], Dennis Moledina[2], Steven G. Coca[3], Jiashu Xue[1], James Kaufman[4], Vernon Chinchillil[5], Paul L. Kimmel[6], T Alp Ikizler[7], Chi-yuan Hsu[8,9], Tanika Kelly[10], Ana Ricardo[10], Jonathan Himmelfarb[11], Chirag R. Parikh[1], for the Assessment, Serial Evaluation, and Subsequent Sequelae in AKI (for the ASSESS-AKI, the TRIBE-AKI, and the Kidney Precision Medicine Project) Consortia

Affiliation:
1. Division of Nephrology, Johns Hopkins University School of Medicine, Baltimore, MD
2. Section of Nephrology, Department of Medicine, Yale School of Medicine, New Haven, CT
3. Division of Nephrology, Department of Medicine, Icahn School of Medicine at Mount Sinai, New York, New York
4. Division of Nephrology, New York University Grossman School of Medicine and VA New York Harbor Healthcare System, New York, NY
5. Department of Statistics, Pennsylvania State University College of Medicine, Hershey, PA
6. Department of Medicine, George Washington University, Washington DC 20037
7. Division of Nephrology, Vanderbilt University, Nashville, TN
8. Kaiser Permanente Division of Research, Oakland, CA
9. Division of Nephrology, University of California, San Francisco, San Francisco, CA
10. Division of Nephrology, Department of Medicine, University of Illinois Chicago, Chicago, IL
11. Center for Kidney Disease Innovation, Icahn School of Medicine at Mount Sinai, New York, New York

Contents

Supplemental Figure 1 Scatter plot of the effect size of the association of proteins with eGFR in all patients and patients with AKI. 2

Supplemental Figure 2. Urine protein concentrations measured by Olink in participants with biopsy proven ATI vs. healthy reference participants from the NAIKID cohort. 3

Supplemental Figure 3. Urine protein concentrations measured by Somascan in participants with AKI vs. healthy reference participants receiving research kidney biopsies from the KPMP cohort. 4

Supplemental Figure 4. Post- vs. pre-operative urine protein concentrations in participants undergoing cardiac surgery from the TRIBE-AKI cohort. 5

Supplemental Figure 5. Low gene expression for 114 urine proteins associated with longitudinal eGFR decline in participants from the KPMP cohort. 6

Supplemental Figure 6. Individual gene expression for urine proteins belonging to the M1 cluster in participants from the KPMP cohort. 7

Supplemental Figure 7. Individual gene expression for urine proteins belonging to the M2 cluster in participants from the KPMP cohort. 8

Supplemental Figure 8. Individual gene expression for urine proteins belonging to the M3 cluster in participants from the KPMP cohort. 9

Supplemental Table 1. Characteristics of participants undergoing cardiac surgery from the TRIBE-AKI cohort. 10

Supplemental Acknowledgements for Consortium Details 11

Supplemental Figure 1 Scatter plot of the effect size of the association of proteins with eGFR in all patients and patients with AKI.

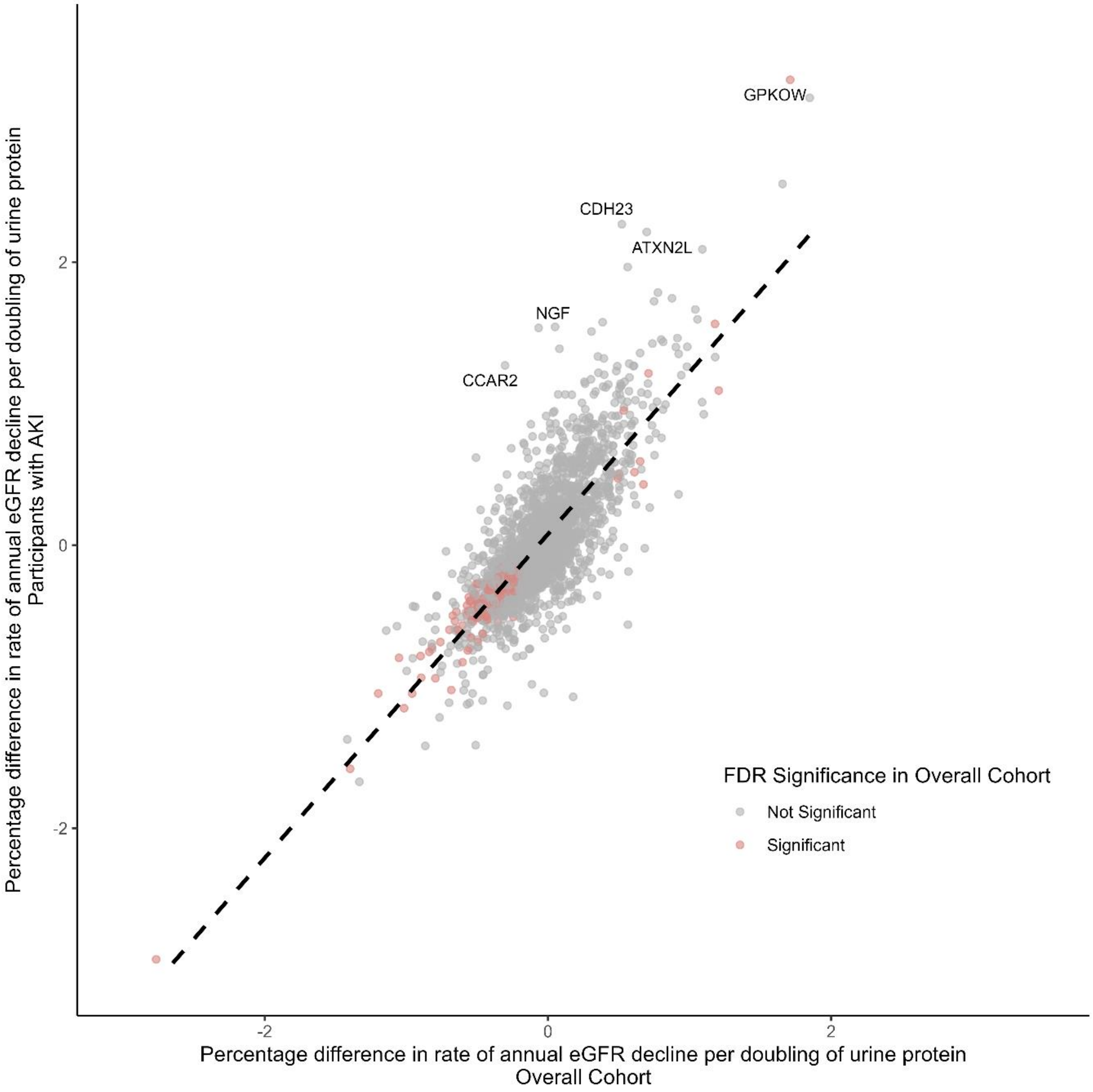

Protein associations with longitudinal eGFR in Overall Cohort (x-axis) vs Participants baseline AKI group (y-axis). Black dashed lineis the identity line; significant proteins in the Overall Cohort represented by red dots (FDR> 0.05).

Supplemental Figure 2. Urine protein concentrations measured by Olink in participants with biopsy proven ATI vs. healthy reference participants from the NAIKID cohort.

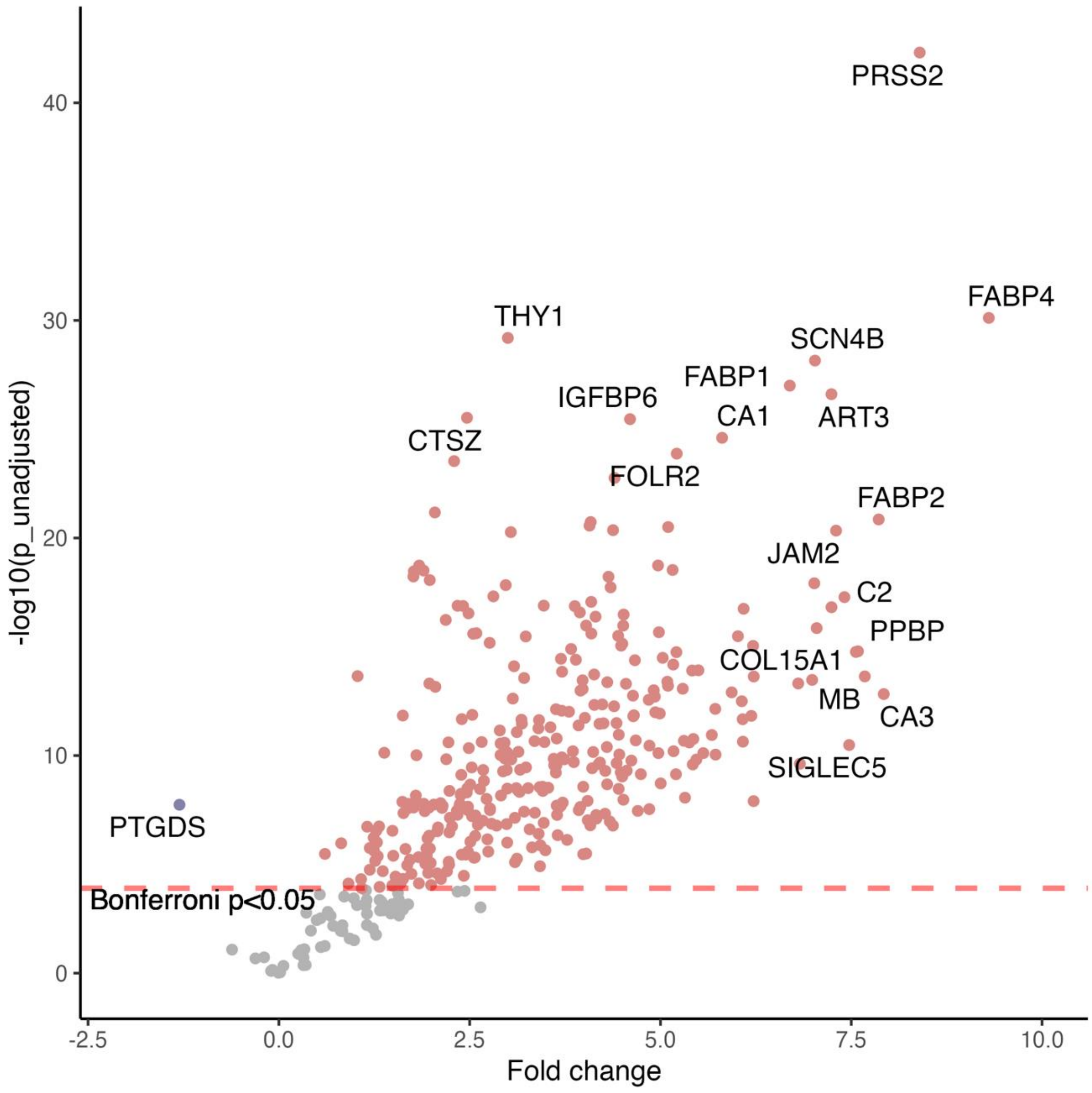

The top 10 proteins in the order of significance for both directions and the top 10 proteins in the order of effect size for proteins higher in patients with ATI are labeled with gene names.

Supplemental Figure 3. Urine protein concentrations measured by Somascan in participants with AKI vs. healthy reference participants receiving research kidney biopsies from the KPMP cohort.

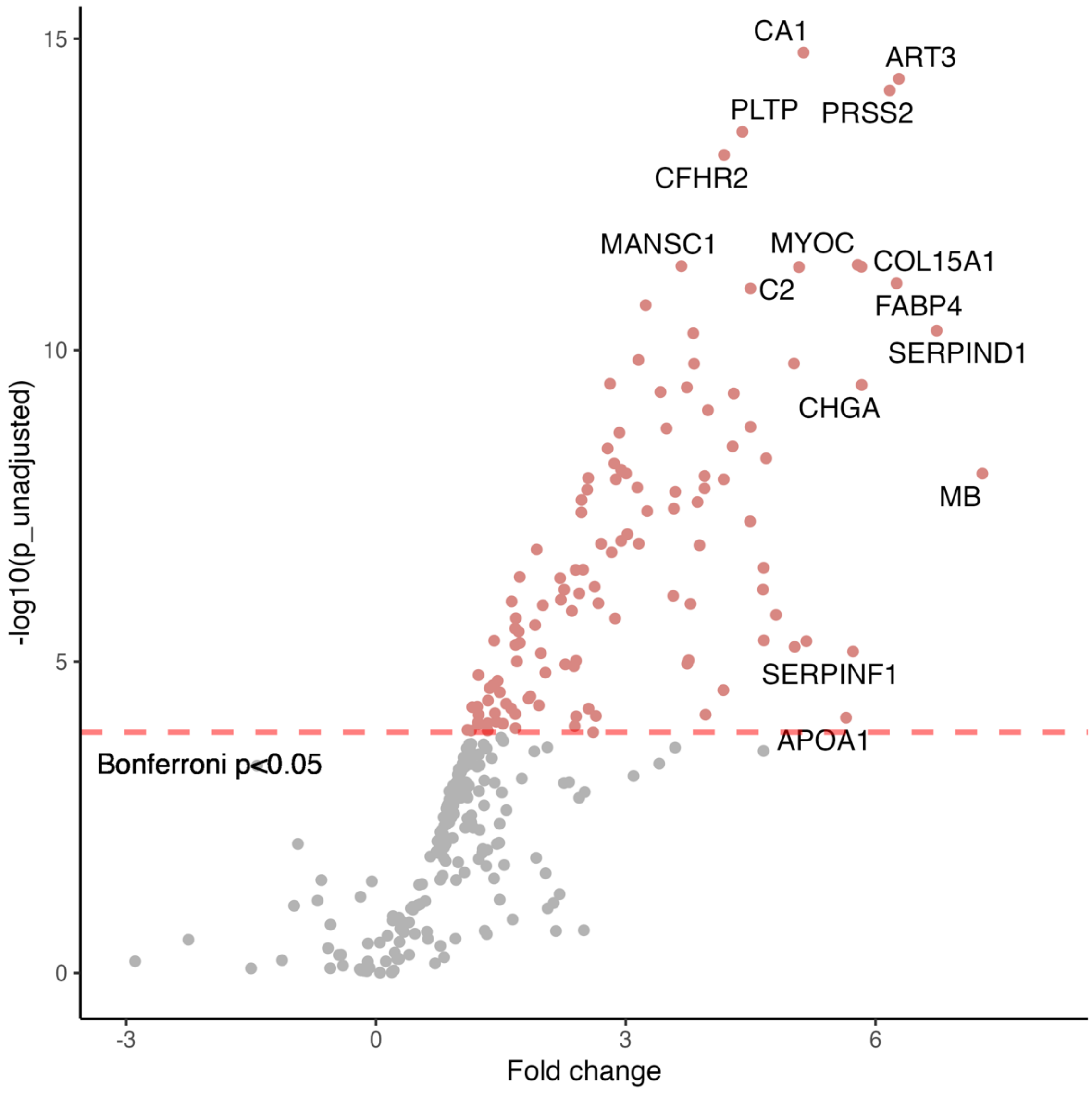

The top 10 proteins in the order of significance and the top 10 proteins in the order of effect size for proteins higher in patients with AKI are labeled with gene names.

Supplemental Figure 4. Post- vs. pre-operative urine protein concentrations in participants undergoing cardiac surgery from the TRIBE-AKI cohort.

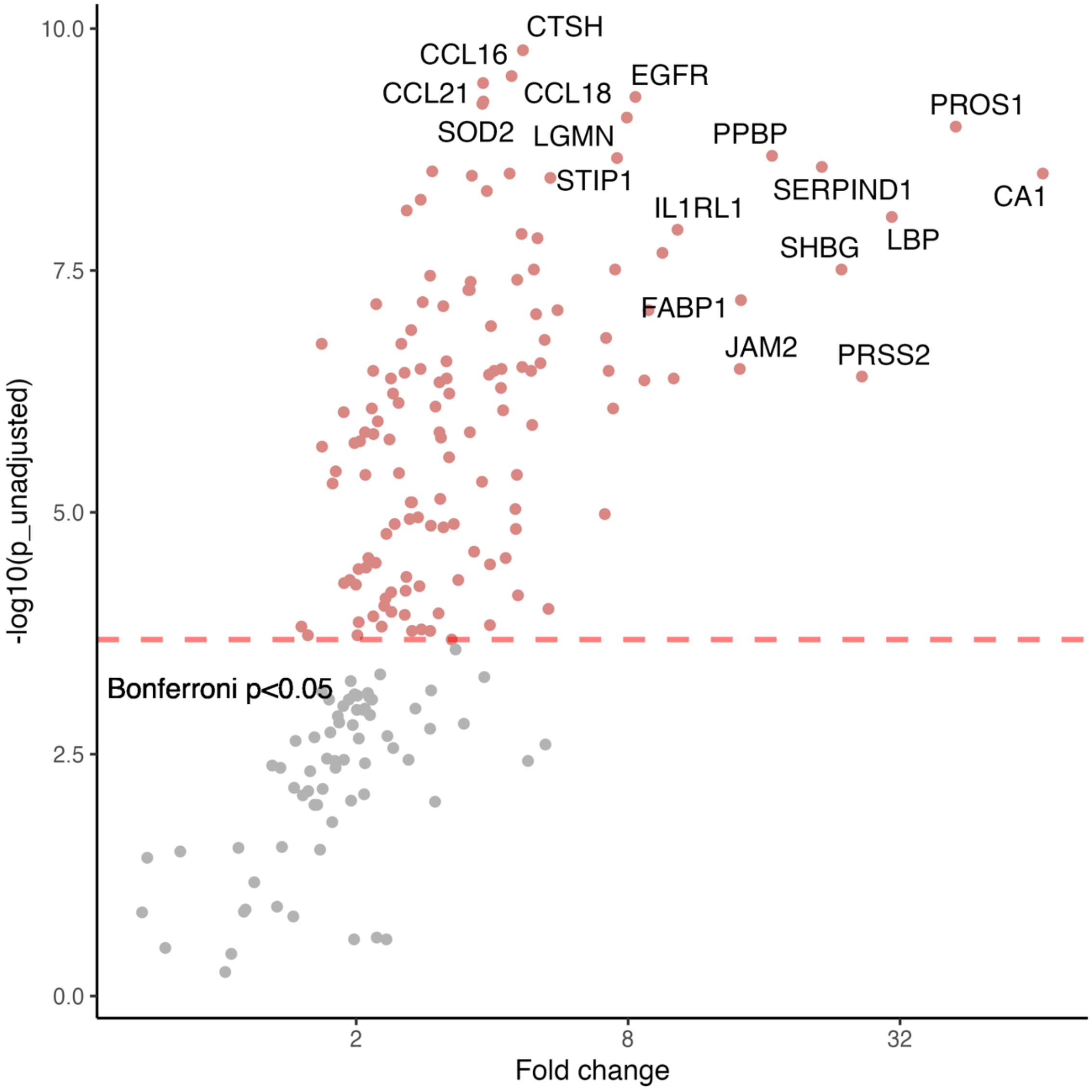

The top 10 proteins in the order of significance and the top 10 proteins in the order of effect size for proteins higher after cardiac surgery are labeled with gene names.

Supplemental Figure 5. Low gene expression for 114 urine proteins associated with longitudinal eGFR decline in participants from the KPMP cohort.

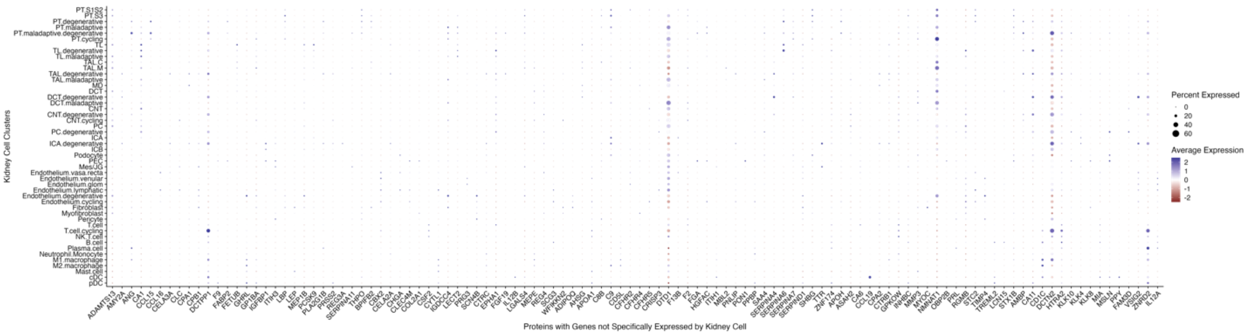

Supplemental Figure 6. Individual gene expression for urine proteins belonging to the M1 cluster in participants from the KPMP cohort.

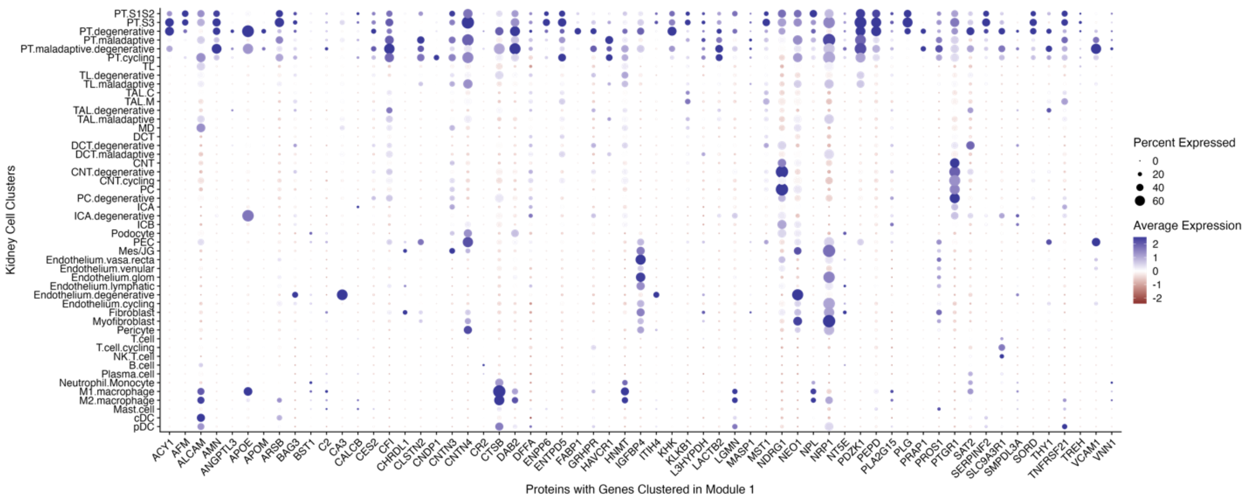

Supplemental Figure 7. Individual gene expression for urine proteins belonging to the M2 cluster in participants from the KPMP cohort.

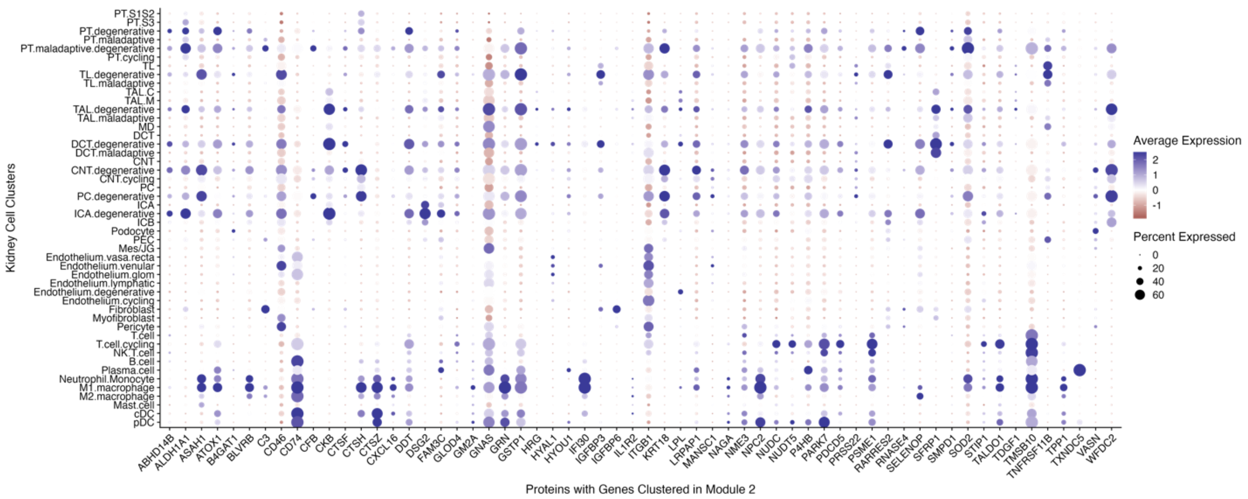

Supplemental Figure 8. Individual gene expression for urine proteins belonging to the M3 cluster in participants from the KPMP cohort.

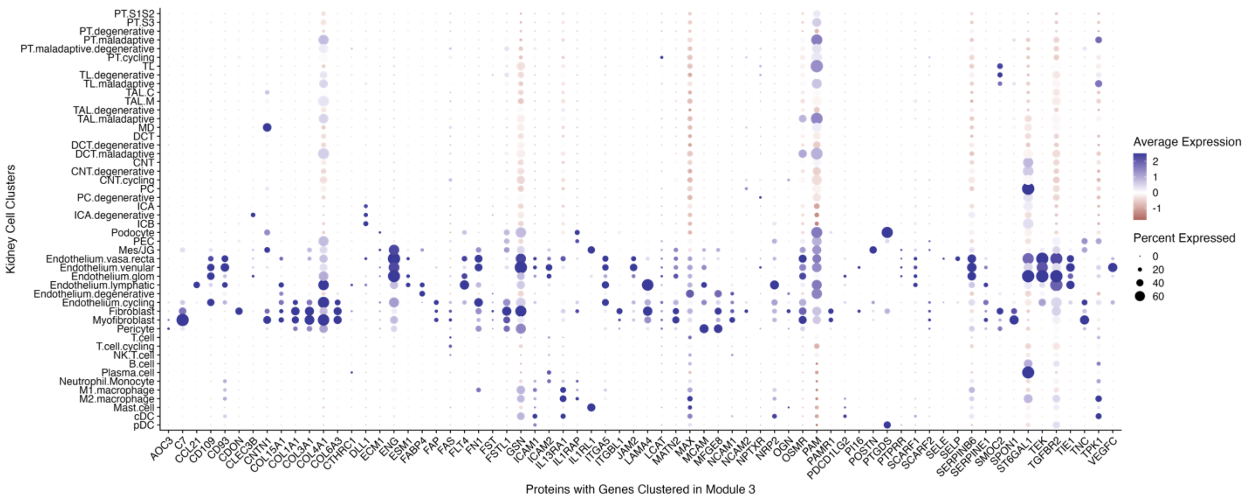

Supplemental Table 1. Characteristics of participants undergoing cardiac surgery from the TRIBE-AKI cohort.

**Characteristics†**		**AKI Progressor (N=27)**	**AKI Non-progressor (N=27)**
**Age, years**		71 [56, 84]	72 [54, 84]
**Sex**	**Male**	24 (88.9%)	24 (88.9%)
	**Female**	3 (11.1%)	3 (11.1%)
**Self Identified White or Caucasian Race**		27 (100%)	27 (100%)
**Hypertension**		25 (92.6%)	24 (88.9%)
**Diabetes**		13 (48.1%)	11 (40.7%)
**Surgery Status**			
Elective		19 (70.4%)	25 (92.6%)
Urgent		8 (29.6%)	2 (7.4%)
**Length hospital stay**		17.0 [5.00, 197]	6.00 [4.00, 81.0]
**Baseline Serum Creatinine (mg/dL)**		1. 20 [0.70, 2.40]	1.00 [0.68, 2.10]
**AKI Stage**			
**Stage 1**		0 (0%)	25 (92.6%)
**Stage 2**		14 (51.9%)	2 (7.4%)
**Stage 3**		13 (48.1%)	0 (0%)

† Summary statistics are presented in median [IQR] and n(%) unless otherwise indicated.

Supplemental Acknowledgements for Consortium Details

The KPMP Consortium: In addition to KPMP Consortium members who are authors (Y.W., S.M., D.G.M., W.O., P.M.P., L.G.C., and C.R.P.), the following KPMP Consortium members are collaborators who have contributed to study design, and participant enrollment: Mohamed G. Atta[1], Mitali Barik[1], Maria Chilo Bejarano[1], Celia P. Corona-Villalobos[1], Derek M. Fine[1], Jose M Monroy-Trujillo[1], Avi Z. Rosenberg[1], C. John Sperati[1], Noralinda B. Viloria[1], Tifanny Budiman[2], Lloyd G Cantley[2], Vijayakumar R Kakade[2], Gilbert W. Moeckel[2], Melissa M. Shaw[2], Jeffrey M Turner[2], Angela M. Victoria-Castro[2], F. Perry Wilson[2], Evren U Azeloglu[3],Kirk N Campbell[3], Lili Chan[3], Marina de Cos[3], Lorraine Evo-Ortega[3], Lili Gai[3], Ronald E. Gordon[3], Mark L Green[3], Ritu Gupta[3], Jens Hansen[3], Jonathan Haydak[3], John Cijiang He[3], Carol R. Horowitz[3], Ravi Iyengar[3], Gina Koch[3], Patricia Kovatch[3], Brandon G Larson[3], Sean Lefferts[3], Patricia Kovatch[3], Kristin Meliambro[3], Girish N Nadkarni[3], Timothy D. Quinn[3], Tejas Rao[3], Daniel Stalbow[3], Isaac E Stillman[3], Joji Tokita[3], Stephen C Ward[3], Samuel Mon-Wei Yu[3], Jamie L Allen[7], Madeline E. Colley[7], Yarieli Cuevas-Rios[7], de Caestecker M.P. [7], Ruining Deng[7], Katerina V. Djambazova[7], Martin Dufresne[7], Melissa A Farrow[7], Agnes B. Fogo[7], Yuankai Huo[7], Audra M. Judd[7], Angela R.S. Kruse[7], Jeffrey M. Spraggins[7], Zoltan G. Laszik[8], Bui, James T[9], Eunice Carmona-Powell[9], Monica L. Fox[9], Ron C. Gaba[9], James P. Lash[9], Natalie Meza[9], Arabela Quiroga[9], Devona Redmond[9] , Amada Renteria[9], Aaron Scroggins[9], Suman Setty[9], Kim Silva[9], Anand Srivastava[9], Michael Tanious[9], Stewart H. Lecker[12], Alexander Morales[12], Mark E. Williams[12], Stephanie J. Aw[13], Laurence H Beck Jr[13], Marie Florence Calixte[13], Kifle Gebre[13] , Molly C Geraghty[13], Joel M Henderson[13], Courtney Huynh[10], Astrid Larson[13], Minxin Lu[13], Keyvona Moultrie[13], Narasimhan, R. [13], Florencia A. Rojas-Miguez[13], Insa M Schmidt[13], Ashish Upadhyay[13], Ashish Verma[13], Sushrut S. Waikar[13], Yan Zhou[13], Nir Hacohen[14], Mark P. Aulisio[15], William S. Bush[15], Yijiang Chen[15], Dana C. Crawford[15], Crystal A Gadegbeku[16], Leal Herlitz[16], Vivian Jeffers[16], Michael Kuperman[16], Marina Markovic[16], Charles O′Malley[16], John F. O′Toole[16], Emilio D Poggio[16], Teresa Randle[16], John R. Sedor[16], Dianna Sendrey[16], Kassandra Spates-Harden[16], Jonathan J Taliercio[16], Paul S. Appelbaum[17], Jonathan Barasch[17], Andrew S. Bomback[17], Vivette. D′Agati[17], Krzysztof Kiryluk[17], German Varela[17], Joana P. Gonçalves[18], Roy Lardenoije[18], Lukasz G. Migas[18], Raf Van de Plas[18], Laura Barisoni[19], Li Xiang[19], Bangchen Wang[19], Anant Madabhushi[20], Andrew Janowczyk[20], Charlotte Boys[21], Robin Fallegger[21], Leonie Küchenhoff[21], Julio Saez-Rodriguez[21], Seth Winfree[21], Theodore Alexandrov[21], Dongwon Lee[22], Jia-Yun Chen[22], Nils Gehlenborg[22], Mark S. Keller[22], Jia-Ren Lin[22], Seymour Rosen[22], Sandro Santagata[22], Mahla Asghari[23], Tarek M. El-Achkar[23], Daria Barwinska[23] , William S. Bowen[23], Andreas Bueckle,

Ying-Hua Cheng[22], Pierre c. Dagher[22], Michael T Eadon[23], Michael Ferkowicz[23], Debora Gisch[23], Danielle Janosevic[23], Katherine J. Kelly[23], Ricardo Melo Ferreira[22], Azuma Nanamatsu[23], Marcelino Rivera[23], Angela R. Sabo[23], Mohammad A. Sohail[23], Jennifer Stashevsky[23], Timothy A. Sutton[22], Curtis Warfield[23], James C. Williams, Jr.[23], Stephanie Wofford[23], Katy Börner[24], Bruce W. Herr II[24], Ellen M. Quardokus[24], Elizabeth G. Record[24], Pottumarthi V Prasad[25], Gek Cher Chan[26], Samir V Parikh[27], Brad H. Rovin[27], Christopher R Anderton[28], Brittney L. Gorman[28], Jessica Lukowski[28], Ljiljana Paša-Tolić[28], Dusan Velickovic[28], Xi, Chen[29], Weiguang Mao[29], Rachel S. G. Sealfon[29], Ksenia Sokolova[29], Olga G Troyanskaya[29], Blue B. Lake[30], Ari Pollack[31], David H. Beyda[32], Erika R Bracamonte[32], Frank C. Brosius[32], Baltazar Campos[32], Austin Derma[32], Griselda Gamez[32], Ana Celina[32], Raymond Scott[32], Bijin Thajudeen[32], Rebecca Tsosie[32], Gregory Woodhead[32], Kun Zhang[33], Milda R. Saunders[34], Ashley R. Burg[35], Hsieh EWY[36], Joshua M. Thurman[36], Samuel Border[37], Manoj Kumar Galla[37], Harshit Lohaan[37], Nicholas Lucarelli[37], Sayat Mimar[37], Ahmed Naglah[37], Anindya S. Paul[37], Pinaki Sarder[37], Fadhl Alakwaa[38], Francesca Annese[38], Heather K. Ascani[38], Ulysses G. J. Balis[38], Markus Bitzer[38], Victoria M. Blanc[38], Nikole Bonevich[38], Ninive Conser[38], Nathan Creger[38], Dawit Demeke[38], Rachel Dull[38], Sean Eddy[38], Josh Hartley[38], John Hartman[38], Yongqun He[38], Jeffrey B. Hodgin[38], Wenjun Ju[38], Matthias Kretzler[38], Chrysta C Lienczewski[38], Laura H. Mariani[38], Phillip J. McCown[38], Rajasree Menon[38], Abhijit S. Naik[38], Viji Nair[38], Edgar A. Otto[38], Rebecca Reamy[38], Michael P. Rose[38], Jennifer A. Schaub[38], Haneen Tout[38], Zach Wright[38], Oyedele A. Adeyi[39], Alison Bunio Alvear[39], M. Luiza Caramori[39], Alyson Coleman[39], Donna D'Souza[39], Yanli Ding[39], Drawz PE[39], Siobhan M. Flanagan[39], Ann Gentry[39], Tasma Harindhanavudhi[39], Dori Henderson[39], Christopher J. Jones[39], Susan Klett[39], Sisi Ma[39], Patrick H. Nachman[39], Oluwatosin Oluwole[39], Elizabeth A. Rogersv[39], Sami Safadi[39], Sandeep Sharma[39], Michelle L. Snyder[39], Susan M. Wolf[39], Zoe Wright[39] Peter R. Bream, Jr. [40], Anne Froment[40], J Charles Jennette[40], Jennifer L. Jones[40], Nicole Keefe[40], Sora Lee[40], Priya Mody[40], Vanessa Moreno[40], Amy K. Mottl[40], Prabir Roy-Chaudhury[40], Saad Mohammed Shariff[40], Alexander Villalobos[40], Evan M. Zeitler[41], Raghavan Murugan[41], Paul M. Palevsky[41], Parmjeet Randhawa[41], Tina Vita[41], Bhupendra Kumar Gurung[42], Soumya Maity[42], NAGARJUNACHARY RAGI[42], Kumar Sharma[42], manjeri venkatachalam[42], Guanshi Zhang[42], Shiqi Zhang[42], Qi Cai[43], Catherine Campbell[43], Choudhary Moaz[43], Allen R Hendricks[43], Sanjeeva P. Kalva[43], Asra Kermani MD[43], Shihong Ma[43], Meredith C McAdams[43], R. Tyler Miller[43], Jiten Patel[43], Boris S. Patlis[43], Samuel Rice[43], Robert D Toto[43], Miguel A. Vazquez[43], Nancy Wang[43], CE Alpers[44], Ashley C Berglund[44], Brooke Berry[44], Kristina N Blank[44],

Keith D. Brown[44], Jonas M Carson[44], Ian H. de Boer[44], Matthew Dekker[44], Ashveena L Dighe[44], Frederick Dowd[44], Andrew N Hoofnagle[44], Nichole M. Jefferson[44], Cienn[44], N. Joyeux[44], Richard A. Knight[44], Christine P Limonte[44], Robyn L. McClelland[44], Jimmy Phuong[44], Alexa Plisiewicz[44], Kasra A Rezaei [44], Glenda V. Roberts [44], Kelly D. Smith[44], Jaime Snyder[44], Christy Stutzke[44], Katherine R. Tuttle[44], Ruikang Wang[44], Artit Wangperawong[44], Bessie A. Young[44], Jeannine Basta[45], Joseph P. Gaut[45], Reetika Ghag[45], Sanjay Jain[45], Madhurima Kaushal[45], Amanda Knoten[45], Asmita L[45], Amy McMurray[45], Brittany C Minor[45], Michael Rauchman[45], Stephanie Reinert[45], Bo Zhang[45]

[12]Beth Israel Deaconess Medical Center, Harvard Medical School, Boston, MA 02215, USA.

[13]Boston University School of Medicine, Boston Medical Center, Boston, MA 02118, USA.

[14]Broad Institute of MIT and Harvard, Cambridge, MA 02142, USA.

[15]Case Western Reserve University School of Medicine, Cleveland, OH 44106, USA.

[16]Cleveland Clinic, Cleveland, OH 44103, USA.

[17]Vagelos College of Physicians and Surgeons, Columbia University, New York, NY 10032, USA.

[18]Delft Center for Systems and Control, Delft University of Technology, Delft, Netherlands.

[19]Duke University, Durham, NC 27708, USA.

[20]Emory University and Georgia Institute of Technology, Atlanta, GA 30322, USA.

[21]European Molecular Biology Laboratory, Heidelberg, Germany.

[22]Harvard University Medical School, Boston, MA 02115, USA.

[23]Indiana University School of Medicine, Indianapolis, IN 46202, USA.

[24]Indiana University, Bloomington, IN 47405, USA.

[25]Northwestern University, Chicago, IL 60611, USA.

[26]National University Hospital of Singapore

[27]Ohio State University, Columbus, OH 43210, USA.

[28]Pacific Northwest National Laboratories, Richland, WA 99354, USA.

[29]Princeton University, Princeton, NJ 08544, USA.

[30]San Diego Institute of Science, Altos Labs, San Diego, CA 92121, USA.

[31]Seattle Children’s Hospital, Seattle, WA 98105, USA.

[32]University of Arizona, Tucson, AZ 85721, USA.

[33]University of California San Diego, San Diego, CA 94143, USA.

[34]University of Chicago, Chicago, IL 60637, USA.

[35]University of Cincinnati, Cincinnati, OH 45221, USA.

[36]University of Colorado, Denver, CA 94143, USA.

[37]University of Florida, Gainesville, FL 32611, USA.

[38]University of Michigan, Ann Arbor, MI 48109, USA.

[39]University of Minnesota, Minneapolis, MN 55455, USA.

[40]University of North Carolina, Chapel Hill, NC 27599, USA.

[41]University of Pittsburgh, Pittsburgh, PA, 15260. USA.

[42]University of Texas Health Science Center at San Antonio, San Antonio, TX, USA.

[43]University of Texas Southwestern, Dallas, TX 78229, USA.

[44]University of Washington, Seattle, WA 98195, USA.

[45]Washington University St. Louis, St. Louis, MO 63130, USA.

ASSESS-AKI Investigators: In addition to ASSESS-AKI Consortium members who are authors (DM, SGC, JK, VC, PLK, TAI, CH, JH, CRP), the following ASSESS-AKI Consortium members are collaborators who have contributed to study design, , and study participant enrollment: Nasrollah Ghahramani[5]; W. Brian Reeves[5]; Alan Go[8,9]; Kathleen Liu[8,9]; Raymond Hsu[8,9]; Thida Tan[8,9]; Juan D. Ordonez[8,9]; Sijie Zheng[8,9]; Edward D. Siew[7]; Julia B. Lewis[7]; Lorraine Ware[7]; Amit X. Garg[46,47,48]; Prasad Devarajan[35]; Michael Zappitelli[49]; Jonathan Himmelfarb[44]; Mark Wurfel[44].

[46]ICES, Toronto, ON, Canada.

[47]Department of Epidemiology and Biostatistics, Western University, London, ON, Canada.

[48]Division of Nephrology, Department of Medicine, Western University, London, ON, Canada

[49]Division of Pediatric Nephrology, Department of Pediatrics, Hospital for Sick Children, Toronto, ON, Canada

TRIBE-AKI Investigators: In addition to TRIBE-AKI Consortium members who are authors (SGC, CRP), the following TRIBE-AKI Consortium members are collaborators who contributed to study design and study participant enrollment: Michael G Shlipak[9]; Jay L Koyner[34]; Charles L Edelstein[36]; Prasad Devarajan[35]; Michael Zappitelli[49]; Catherine D Krawczeski[35]; Cary S Passik[50, 51]; Madhav Swaminathan[19]; and Amit X Garg[46,47,48].

[50]Department of Cardiothoracic Surgery, Danbury Hospital, CT

[51]University of Vermont College of Medicine, Burlington, Vermont
